# Correction for Zhao et al., “IDO1 promotes CSFV replication by mediating tryptophan metabolism to inhibit NF-κB signaling”

**DOI:** 10.1128/jvi.01094-25

**Published:** 2025-09-30

**Authors:** Feifan Zhao, Yaoyao Huang, Junzhi Ji, Xueyi Liu, Xiaowen Li, Linke Zou, Keke Wu, Xiao di Liu, Sen Zeng, Xinyan Wang, Wenshuo Hu, Yiwan Song, Zhimin Lu, Bolun Zhou, Peng Li, Weijun Wang, Mingqiu Zhao, Jinding Chen, Lin Yi, Shuangqi Fan

## AUTHOR CORRECTION

Volume 98, no. 7, e00458-24, 2024, https://doi.org/10.1128/jvi.00458-24. Page 6: Figure 3B and E should appear as shown in this correction.

**Fig 3:**
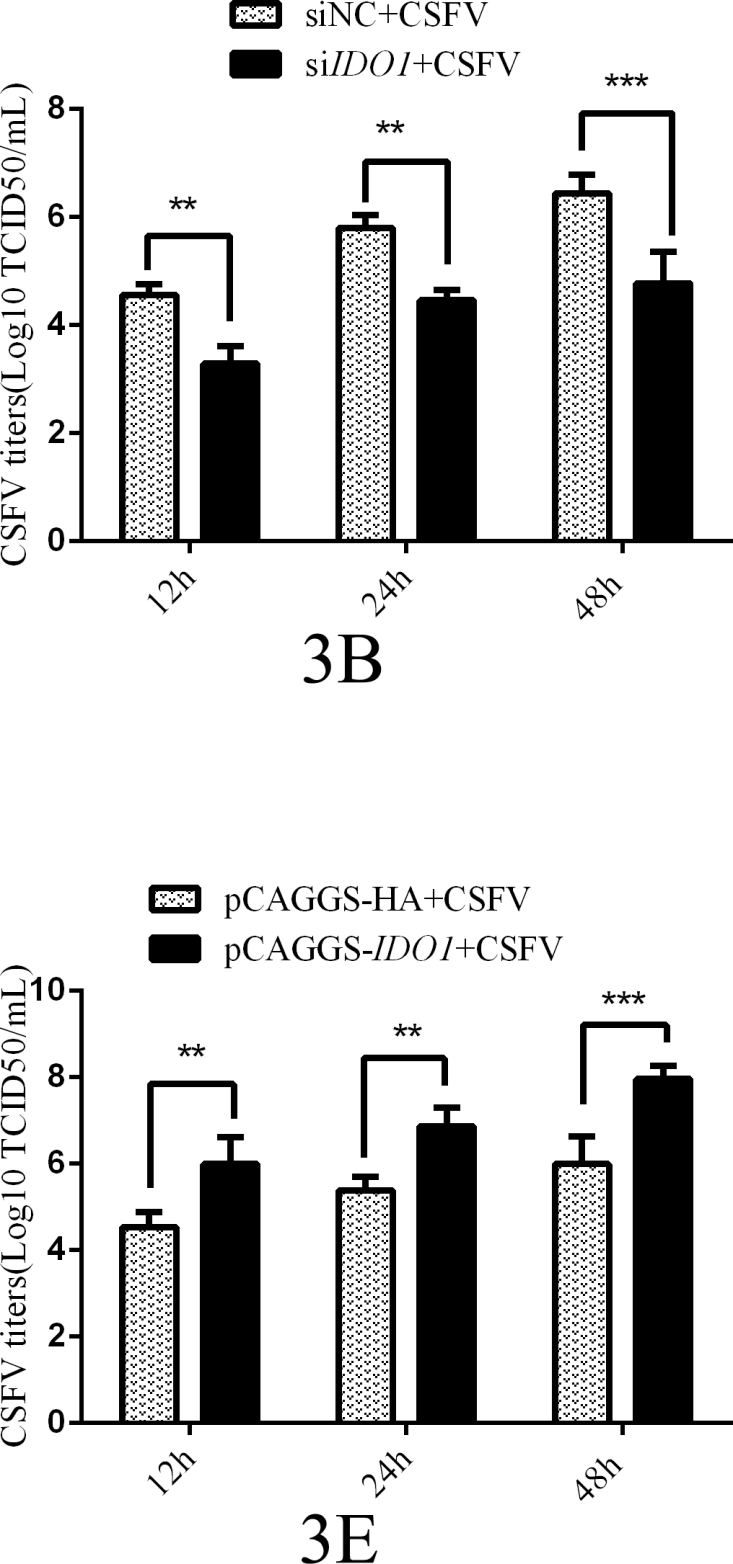


Page 6: In Fig. 3A and C, “SCFV” should read “CSFV.”

We apologize for these errors, which did not change the final result.

